# 1158. Pediatric Group A Streptococcal Peritonitis: A Single-Center Eleven Patient Case Series

**DOI:** 10.1093/ofid/ofab466.1351

**Published:** 2021-12-04

**Authors:** Nicole L Pershing, Scott Eldredge, Jack E Burgeson, David Dansie, Katie Russell, Kent Korgenski, Krow Ampofo, Elizabeth Vukin

**Affiliations:** 1 University of Utah, Salt Lake City, Utah; 2 Primary Childrens Hospital, Salt Lake City, Utah

## Abstract

**Background:**

Pediatric group A streptococcal peritonitis (GASP) is a rare but serious infection, with few cases reported in the literature. Utah has an unusually high incidence of invasive GAS (iGAS) disease, but the frequency and characteristics of pediatric GASP are unknown.

**Methods:**

We performed a retrospective chart review to identify GASP in Utah children from 2000-2019. GASP was defined as isolation of GAS from peritoneal fluid or blood and clinical signs of peritonitis.

**Results:**

: Eleven children with GASP were identified, with slight female predominance (n=6). Median age was 6 years; males were significantly younger than females (1.4 versus 7.2 years, *p*=0.01). GAS was isolated from 4 of 8 blood and 8 of 11 peritoneal cultures obtained. Peritoneal fluid PCR was positive for GAS in one patient. Ten patients underwent laparotomy. Peri-appendiceal inflammation prompted appendectomy in 7 patients; only one had pathologic findings of acute appendicitis. Four patients developed streptococcal toxic shock syndrome and 7 required intensive care. Non-white race (n=4) and lack of appendectomy (n=5) were associated with more severe outcomes. Median antibiotic duration was 27 days. Median hospitalization was 8 days. All patients survived.

Figure 1. Schematic representation of GAS peritonitis patient clinical course.

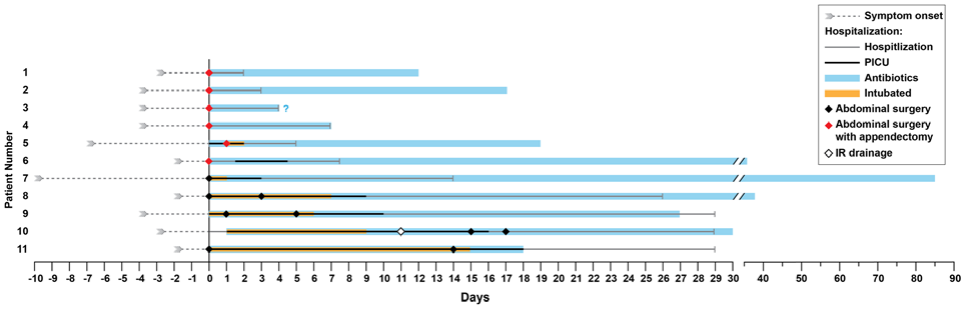

Each patient is represented by a single line. Duration of symptoms prior to hospitalization, as well as duration of hospitalization (day 0 representing admission), intensive care, antibiotic administration, and timing of procedural interventions are noted. Duration of antibiotics after discharge for patient 3 was unable to be verified, as indicated by a question mark. Hospitalization, general pediatric hospital care. PICU, pediatric intensive care unit. IR, interventional radiology.

**Conclusion:**

We present the largest pediatric case series of GASP to date. Diagnostic hallmarks included gastrointestinal symptoms, fever, systemic inflammation, and peritoneal enhancement without an abdominal source. Peri-appendiceal inflammation was common, although acute appendicitis was rare, and appendectomy was associated with a less severe course. GASP should be considered in patients with acute abdominal processes given increasing incidence of iGAS infections.

**Disclosures:**

**All Authors**: No reported disclosures

